# Supplementary material for: Vascularization of Poly-ε-Caprolactone-Collagen I-Nanofibers with or without Sacrificial Fibers in the Neurotized Arteriovenous Loop Model
Source: Cells. 2022 Nov 25;11(23):3774. doi: 10.3390/cells11233774 (PMC9736129; doi:10.3390/cells11233774)
Supplement: Supplementary file 1 [file cells-11-03774-s001.zip › Supplementary Information.pdf]

Supplementary Information

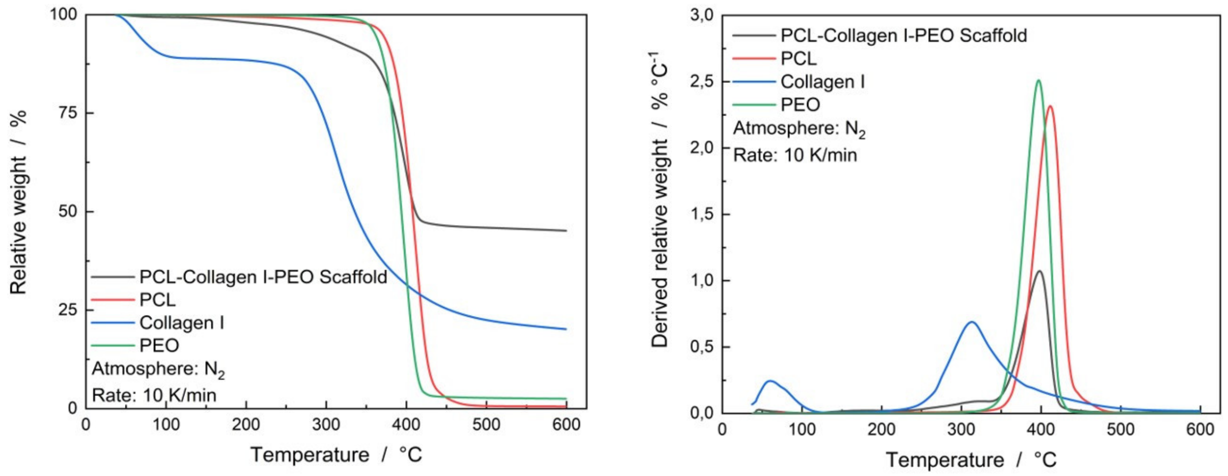

Figure S1: Standard and derived TGA curves of the as-spun PCL-collagen I-PEO scaffolds as well as the individual components PCL, collagen I, and PEO. The decomposition temperatures of PCL and PEO are too similar to detect PEO residues in the nanofibrous scaffolds.

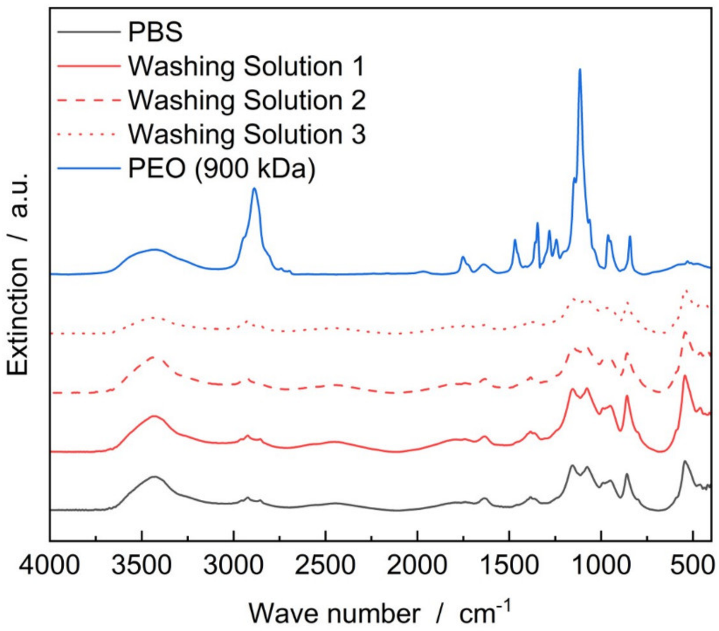

Figure S2: FT-IR analysis of PBS washing solutions of PCL-collagen I-PEO-nanofiber scaffolds, clear PBS solution and PEO, showing identical patterns of PBS washing solutions as the clear PBS solution and none of the characteristic peaks as PEO.

Video S1: Multiphoton microscopic images in multiple slices allow a three-dimensional processing of all layers of PC4 construct
